# Reviews and Book Notices

**Published:** 1882-03

**Authors:** 


### Reviews and Book Notices.

ARTICLE XI. — AN ELEMENTARY TREATISE ON PRACTICAL CHEMISTRY AND QUALITATIVE INORGANIC ANALYSIS. By FRANK CLOWES, D.S.C., London. With Illustrations. From the Third English Edition. 12mo, cloth, pp. 372. Philadelphia: H. C. Lea's Son & Co. 1881.

The author says in his preface: "The book is especially intended to furnish a course of instruction in practical chemistry in the laboratories of our public and other schools." This aim has been successfully attained. The treatise is also adapted for medical and university students, and is essentially practical. The faults of such a work consist in separating qualitative from quantitative analysis, which should be studied together preferably, and in confining its domain to inorganic substances only. Thus a student might be proficient in his study, yet he would not deserve the title of analytical chemist, after thus cutting the matter to one-fourth.

This is a fault manifested in many scientific books, however, and is a division of labor which may have its usefulness, after all, especially if other branches have been partially looked into by the specialist.

The book is well printed, and the illustrations (of instruments mainly) are well executed. A large part of the volume consists of tables, which, although presenting the subject in a condensed and comparative form, are very intricate, and must be rather unattractive for most students.

ARTICLE XII.—PHYSIOLOGICAL LABORATORY, HARVARD MEDICAL SCHOOL, Boston. Collected Papers, 1873–1879. For Private Circulation. 8vo, cloth, about 150 pages. Illustrated with Photographs, Wood-cuts and Diagrams.

Many valuable physiological experiments are described in this book, which begins with a prize essay by C. H. Williams, A. B., "Experiments on the Action of Bile in promoting the Absorption of Fats," and one "On Intestinal Digestion," by G. M. Garland, which also received the prize of the Boylston Medical Society.

In a valuable series of experiments, O. L. Walton shows that the epiglottis is of little account in deglutition, but that its main function is in regulating speaking and singing.

Several contributions by Dr. H. P. Bowditch are contained in the same volume, which will prove more valuable to authors and professors than to practitioners.

ARTICLE XIII.—HANDBOOK OF CHEMICAL PHYSIOLOGY AND PATHOLOGY, WITH LECTURES UPON NORMAL AND ABNORMAL URINE. By VICTOR C. VAUGHAN, M.D., PH.D. Third Edition. Revised and Enlarged. Contains an Appendix of Twenty-six Plates, illustrating the Chemistry of the Urine. 8vo, cloth, pp. 351. Ann Arbor: Ann Arbor Printing and Publishing Co. 1880.

This manual contains but very little original matter, yet it presents a tolerably good résumé of the researches on which this new science is based, and is entirely practical.

The pathological part is mainly concerned with urine analysis, and does not justify the title of the book. In fact, more than half of it treats of the urine. The plates, most of them copied from other text-books, are very good indeed; the text is clear and well printed, and the binding substantial. More investigation into the chemical changes attending fatty degeneration, cancers, infectious diseases, and various tissue metamorphoses, would augment materially the value of such a work for students and practitioners.

ARTICLE XIV. — A HANDBOOK OF VERTEBRATE DISSECTION. By H. NEWELL MARTIN, D.S.C., M.A., M.D., and W. A. MOALE, M.D. Part I. How to Dissect a Chelonian. 12mo, cloth, pp. 94. New York: Macmillan & Co. 1881.

Our readers will probably remember that Dr. Martin, Professor in Johns Hopkins University, was the joint author of the valuable little book known as Huxley's Biology.

The book now under consideration treats of the dissection of a mud turtle, also known as terrapin; and two other volumes, treating respectively of a pigeon and of a rat, will soon follow. A plate of four figures, representing the skull, accompanies this volume, which, in our opinion, ought to have been well illustrated, since anatomy is so difficult a subject, especially for beginners. Two main defects will be noticed on using this book, (*a*) no measurement of organ or of skeleton is referred to, and (*b*), the relations of the anatomical parts of the turtle to the corresponding parts of man, and to those of animals lower in the series, which is not alluded to. The oft-repeated words notice, observe, remark, are of far less value than they seem, for any student undertaking to dissect a mud turtle is usually endowed with a natural faculty of observation. As this is a new departure in anatomical study, however, and intended as a help to students, we believe that it is to be commended; and at the same time, the authors are highly qualified for the present undertaking.

ARTICLE XV.—THE PRINCIPLES OF MYODYNAMICS. By J. S. WRIGHT, M.D., Professor of Surgery, and Lecturer on Physical Science at the Long Island College Hospital. 12mo, cloth, pp. 162. Illustrated with Twenty nine Diagrams. New York: Bermingham & Co. 1881.

At first sight, this work recalls the Pleurodynamics of Dr. Garland, and one expects to find in it the *causes* of contractions in muscles, a subject which has given rise to a good deal of discussion of late; or, at least, the weight under which muscles would break, etc.; but there is no dynamics about this book; it is simply the mechanism of lever-action in bones and muscles, illustrated by a great many diagrams. The book may not prove useless, however, for, as the author says in his preface: "The better the surgeon understands the principles of myodynamics, the better he can treat fractures, dislocations and deformities"—other things being equal, of course.

ARTICLE XVI.—THE WILDERNESS CURE. By MARC COOK, Author of "Camp Lon." 12mo, cloth, pp. 153.

The author is not a physician, but his ideas seem well grounded, and are sustained by many prominent physicians in New York, who praise the Adirondack mountains as a most valuable resort for consumptives. This little book is interesting, and well adapted for any visitor to the northern part of New York State. Besides its picturesque scenery, and the great contrast which the Adirondack country offers to the residents of New York City, it is more than doubtful whether it should be recommended as a health resort. A personal investigation of the matter has led the reviewer to believe that no advanced case of phthisis could be benefited by visiting such a barren tract of land, exposed, as it is, to the most sudden changes of temperature. However, the proximity of the Adirondacks to Saratoga ; their wilderness, contrasted with so many large cities all around, probably make it the best summer hunting ground, and a most beautiful country in which to spend a vacation, especially whenever health fails the Eastern student or business man.

ARTICLE XVII. — THE ESSENTIALS OF ANATOMY. By WILLIAM DARLING, M.D., F.R.C.S., and AMBROSE L. RANNEY, A.M., M.D., 8vo, cloth, pp. 629. New York: G. P. Putnam's Sons. 1880.

This voluminous "Essentials" is intended as an accompaniment of Gray's Anatomy, or other large text-book, whereby the memorizing of anatomical facts and names can be facilitated. Thus, it contains tables enumerating the muscles cut in various amputations; colored diagrams to help the student in remembering the course of the circulation ! the total number of muscles supplied by any special nerve in each particular muscular region, and a number of other similar intricacies, which make the study of anatomy so difficult, and necessarily so impractical.

It is well that the authors, in their preface, anticipated the poor reception which their work would receive at the hands of the critics. If they had condensed their material into a book of 200 pages, by cutting the blanks as far as practicable, and had it printed in smaller type, most students would probably welcome it. But as it is, it will remain better known as the cause of an important law-suit regarding the rights of a lecturer to his lectures, than as a valuable volume.

ARTICLE XVIII.—THE THERAPEUTICS OF GYNÆCOLOGY AND OBSTETRICS. Second edition, thoroughly revised and greatly enlarged. Edited by W. B. Atkinson, A.M., M.D. 8vo, cloth, pp. 571. $4. Philadelphia: D. G. Brinton. 1881.

The number of authors quoted being 422, and the number of formulæ 500, it is clear that the subject is exhaustively treated. In fact, this is a sort of encyclopædia, forming with "Modern Medical Therapeutics" and "Modern Surgical Therapeutics" the best collection of prescriptions ever made. And this is the main recommendation of these works. The book under consideration is especially adapted for those practitioners who are unable to construct their own prescriptions, or too busy to study the late works on Therapeutics; but it would be more likely to mislead students than impart the necessary knowledge of the subjects treated in such a vague manner. It is useless to add that no physician of ability ever copies the formulæ of others; and upon inquiry we generally find that the prescriptions thus purporting to be the favorite formulæ of professor so and so, have been used by him but occasionally, for all physicians vary their medicaments according to the circumstances of the case.

However, the numerous quotations which fill up this volume are entirely modern, and the editor seems to have spared no pains to make the work as useful and as practical as could be.

ARTICLE XIX.—PATHOGENETIC OUTLINES OF HOMŒOPATHIC DRUGS. By Dr. Med. Carl Heinicke, Translated from the German, by Emil Tietze, M.D. 8vo, cloth, pp. 576. New York and Philadelphia: Boericke & Tafel. 1880.

The author remarks that the registration of symptoms in the present work has been arranged according to the *anatomico-phy-*

*siological schema.* Indeed, homœopathy is undergoing quite a metamorphosis of late. However, Dr. Heinicke prides his *school* on the discovery that some inert substances homœopathically pre pared, develop effects of great practical value (in homœopathic practice alone, we suppose). Marble, charcoal, carbonate of magnesia, graphite and quartz, written in Latin, are the substances alluded to. But we may add that they are seldom used by our confreres, who rely more on mercurials, opiates, quinine, and aconite than we regulars sometimes do, as we know from homœopathic physicians themselves.

If the book was not one of the productions of the new school, we should feel obliged to say that it is the silliest work ever written on therapeutics. However, when the author vouches for the millionth dilution of one grain of carbonate of lime, and the shaking of the contents with *two powerful strokes of the arm*, which number used to be ten as patented at first by Hahnemann, we are struck with awe, and veneration. We highly recommend the book to all of the homœopathic fraternity, and expressly recommend them to add the word homœopathic after their name; for this is not the profession of medicine, it is the application of certain drugs to certain symptons, while *we* profess to study and treat diseases on rational principles.

ARTICLE XX.—A TREATISE ON THE DISEASES OF THE NERVOUS SYSTEM. By WILLIAM A. HAMMOND, M.D. With 112 Illustrations. Seventh edition, rewritten, enlarged and improved. 8vo, cloth, pp. 930. New York: D. Appleton & Co. 1881.

Professor Hammond is one of the most remarkable physicians who have ever adorned the profession in America. His immense, original and careful investigations, have obtained for him a reputation which will be everlasting. But his very originality, while it has given him such a reputation in Germany, and the honor of so many quotations in Ziemssen's Cyclopædia, has made him many enemies in this country. In fact, Dr. Hammond seldom misses a chance to come before the public, be it to criticise the attendants of the late president, or to defend Dr. Beard and hypnotism. But this is a necessity of the times, when good writers have to make their own way, and look for a living besides.

We always heard or read Dr. Hammond with interest and pleasure, not to say with enthusiasm. His work on Nervous Diseases as now presented in its new form is entirely one of the best text-books on the matter, and we recommend it highly to students and to practitioners as well. The fact that this is the seventh edition in ten years, would of itself be the best recommendation. Besides strictly nervous diseases, this work also treats of hydrophobia, exophthalmic goitre, syphilis of the nervous system, plumbism, alcoholism, bromism, hydrargism and arsenicism, besides the diseases of the sympathetic nervous system.

ARTICLE XXI.—A PRACTICAL TREATISE ON NASAL CATARRH. By Beverly Robinson, A.M., M.D. 8vo, cloth, pp. 182. New York: William Wood & Co. 1880.

This little monograph, notwithstanding its deficiency in some respects, will be welcomed by the profession. It is brief, carefully prepared, and contains a number of useful hints, directions and prescriptions, which have the advantage of being entirely practical.

Here are some of these formulas. In the way of local applications to the inflamed mucous lining, in acute coryza, the author highly recommends :

℞ Pulv. fol. belladonnæ........gr. xx.
Pulv. morphiæ sulph........gr. ij.
Pulv. gum acaciæ............ad. ℥ss.

M. S. Use with the powder blowers for anterior and posterior nares.

The next is a formula for internal use :

℞ Potass. citratis,
Syr. ipecac.,
Tinct. opii camphor.. .......aa ʒij.
Syrupi acaciæ ... ............ ℥i
Aquæ.........................ad ℥ij

M. S. A teaspoonful every hour and a half or two hours.

As a substitute for some mineral water, to move the bowels, the author gives :

℞ Magnesii sulph............℥ss. to ℥j

Magnesii carbon...........ʒss.

Syrupi limonis............℥ss.

Aquæ menth. piperitæ.....ad ℥j.

M. S. To be taken as a dose.

In chronic coryza, the following powders blown through the nostrils posteriorly and anteriorly are beneficial.

℞ Pulv. iodoform..............ʒij

Pulv. camphoræ.............ʒi.

Pulv. acid tanniæ...........gr. v.

Pulv. g. acaciæ..............ʒij. M.

℞ Hydrarg. chlor. mitis.........ʒi

P. sacch. alb.................ʒij.

P. morphiæ sulph............gr. j.

P. bismuthi subnit...... .....ʒij. M.

ARTICLE XXII.—LECTURES ON THE SURGICAL DISORDERS OF THE URINARY ORGANS; Delivered at the Liverpool Royal Infirmary. By Reginald Harrison, F.R.C.S. Second edition, considerably enlarged. London: J. & A. Churchill. 1880.

These lectures are illustrated with eight lithographs, and thirty-eight wood-cuts. By far the larger part of these lectures have been taken up with stricture of the urethra, and calculi in the bladder, with their numerous complications. These subjects are treated in a masterly manner, and will well repay a careful study by surgeons who have occasion to meet these affections often. The few cases reported, give the work still greater value, and it is needless to remark that it is entirely practical.

We are glad to hear Dr. Harrison raise his voice against a mere splitting instead of removal of the prepuce, when circumcision is necessary. Not only are the *angles* left inconvenient, but they are a disfigurement which will generally remain as an index of former venereal disease.

On the whole, these lectures are quite a valuable contribution to the literature of the subject, and represent a vast amount of personal experience. As to the appearance of the book, the printing and the binding, it is inferior to the average medical book published in the country.

ARTICLE XXIII.—A TREATISE ON FOOD AND DIETETICS. By F. W. Pavy, M.D., F.R.S. Second edition. 8vo, cloth, pp. 402. Forming the August No. of Wood's Library for 1881.

One of the most valuable books in the series. The intrinsic value of four such works more than repay for the money paid on the whole. In fact, although Wood's Library is not composed of the best text books, it nevertheless inaugurates a new era in the publication of medical treatises, and enables the poorest practitioners of medicine to have a complete library at their command for the small sum of little more than one dollar a month.

Dr. Pavy, in his introductory remarks on the dynamic relation of food, brings the subject down to the latest discoveries of modern science, and treats it in a masterly manner, except that he overlooks the "adulteration of food," which is not unimportant in this age, and the means of detecting various adulterations ought to have been mentioned at least.

The numerous analyses of various foods, their work-producing capacity, and various other considerations are of the highest practical value. On the controverted subject of beef-tea, the author expresses himself in these words: "Beef-tea represents a highly nutritive and restorative liquid, with an agreeable flavor," but the true position of Liebig's extracts of meat, is scarcely that of an article of nutrition. If not truly of alimentary value, the preparation, nevertheless, appears to possess stimulant and restorative properties which render it useful in exhausted states of the system.

The chapter on "animal foods sometimes but not ordinarily eaten," is full of curious accounts of cannibalism, of dirt-eating habits, of odd favorite dishes, and things not generally known on that subject; representing a vast amount of literature.

